# Waste Detection System Based on Data Augmentation and YOLO_EC

**DOI:** 10.3390/s23073646

**Published:** 2023-03-31

**Authors:** Jinhao Fan, Lizhi Cui, Shumin Fei

**Affiliations:** 1 School of Electrical Engineering and Automation, Henan Polytechnic University, Jiaozuo 454000, China; 242007020001@home.hpu.edu.cn; 2 Henan Key Laboratory of Intelligent Detection and Control of Coal Mine Equipment, Henan Polytechnic University, Jiaozuo 454000, China; 3 School of Automation, Southeast University, Nanjing 210096, China; smfei@seu.edu.cn

**Keywords:** waste classification, target detection, data augmentation, DCGAN, YOLOv4

## Abstract

The problem of waste classification has been a major concern for both the government and society, and whether waste can be effectively classified will affect the sustainable development of human society. To perform fast and efficient detection of waste targets in the sorting process, this paper proposes a data augmentation + YOLO_EC waste detection system. First of all, because of the current shortage of multi-objective waste classification datasets, the heavy workload of human data collection, and the limited improvement of data features by traditional data augmentation methods, DCGAN (deep convolution generative adversarial networks) was optimized by improving the loss function, and an image-generation model was established to realize the generation of multi-objective waste images; secondly, with YOLOv4 (You Only Look Once version 4) as the basic model, EfficientNet is used as the backbone feature extraction network to realize the light weight of the algorithm, and at the same time, the CA (coordinate attention) attention mechanism is introduced to reconstruct the MBConv module to filter out high-quality information and enhance the feature extraction ability of the model. Experimental results show that on the HPU_WASTE dataset, the proposed model outperforms other models in both data augmentation and waste detection.

## 1. Introduction

With the continuous increase in the urban population, the amount and types of waste are also increasing. Improper disposal of waste not only leads to serious environmental pollution but also causes much waste of resources. According to the forecast of the World Bank Organization, the annual waste produced in the world will increase from 2.01 billion tons in 2018 to 3.4 billion tons in 2050 [1]. How to effectively control growing waste has become a serious social problem. Waste classification is regarded as an important measure to solve the waste dilemma and improve resource utilization in the existing environment.

Manual waste sorting presents several issues, including high workload, low efficiency, and poor sanitary conditions. Therefore, the utilization of intelligent, automated methods in the sorting process can significantly reduce labor costs and enhance the utilization of resources. For a long time, researchers have conducted extensive studies in the field of waste sorting, achieving significant progress in waste image recognition and classification. For instance, Yang et al. [2] proposed an SVM (support vector machine) waste classification system that uses a fixed-size sliding window for input images to extract features and then uses the classifier SVM to classify them with an accuracy of 63%. Tachwali et al. [3] used decision trees to classify bottles based on their chemical composition and color, achieving an accuracy of 83.48%. With the advancement of deep-learning technology, the use of deep-learning methods for waste processing has become a major research direction for scholars. Ma et al. [4] used improvements to the SSD (single-shot multibox detector) feature fusion module, solved the positive and negative sample imbalance problem by focal loss, and used a new backbone extraction network, and the improved model showed a large improvement in accuracy and speed. Mao et al. [5] developed a domestic waste dataset based on the Taiwan region for YOLOv3 training and achieved an accuracy of 92.12%. Zhang et al. [6] proposed a YOLO_WASTE waste classification model, which was trained using migration learning based on the YOLOv4 network and achieved excellent results on a self-built dataset.

Presently, for waste image recognition research, most waste image datasets focus on single waste targets, and the collective amount of multi-objective waste data is limited. To address this issue, this paper designs a data enhancement method for expanding multi-objective waste images, which has a positive impact on improving the performance of waste target detection. Furthermore, a light weight target detection model is proposed to address the challenges of excessive network parameters and model redundancy of traditional target detection algorithms. The model is optimized by introducing an attention mechanism to achieve more accurate and efficient detection in waste detection. The paper’s main contributions can be summarized as follows:

1. Introducing the W distance as the DCGAN loss function and using the improved DCGAN network to generate multi-target waste images to solve the problem of tight training samples and lack of richness during detection work;
2. Using the EfficientNet-b2 as the YOLOv4 backbone network to reduce model parameters and realize the light weight of the algorithm;
3. Reconstructing the MBConv module using the CA attention mechanism to better capture the location information and spatial information of waste targets in the feature extraction process.

## 2. Related Work

### 2.1. Data Augmentation

Deep-learning models can achieve image recognition with high accuracy which cannot be achieved without the support of a large amount of data in the dataset. Data augmentation [7] is a common dataset expansion method that has been used to improve the generalization ability of a model by increasing the number of training samples. There are two categories of data augmentation based on the different ways of generating samples: non-generative data augmentation and generative data augmentation.

#### 2.1.1. Non-Generative Data Augmentation

Non-generative data augmentation primarily comprises two categories of transformations: geometric and non-geometric. Geometric transformations focus on altering image morphology through operations such as flipping, rotating, cropping, scaling, and deforming. Non-geometric transformations, on the other hand, include methods such as adding noise, blur, color transformation, random erasure [8], and the superpixel method. While non-generative data augmentation is a straightforward and widely used technique, overuse of these methods can lead to generating data samples that lack practical value.

#### 2.1.2. Generative Data Augmentation

One of the most popular generative data augmentation methods is the generative adversarial network (GAN) [9] and its derivative models. GAN models are a deep-learning-based data generation method, which is based on game theory and uses unsupervised learning to learn the dataset and generate high-quality data. These methods are employed in many fields, including industry, medicine, and mapping. For example, Chen et al. [10] used Cycle-GAN to generate petrochemical pipeline defect images and expanded their dataset, achieving an average accuracy of 93.10% by training the target detection model. Salehinejad et al. [11] used DCGAN to augment chest radiographs and used a combination of real and generated images to train a deep convolutional neural network to achieve pathological detection of five different types of chest radiographs. Jiang et al. [12] applied the edge-enhancement GAN (EEGAN) to satellite remote sensing imaging with promising results.

### 2.2. Deep-Learning-Based Target Detection Algorithm

Target detection is a computer vision technology proposed for detecting specific objects, which finds extensive use in industrial inspection, aerospace, intelligent monitoring, and many other fields. With the maturity of deep-learning theory, target detection algorithms based on deep learning have rapidly developed. The commonly used target detection algorithms can be divided into two categories: two-stage target detection algorithms (two-stage) and one-stage target detection algorithms (one-stage).

Two-stage target detection algorithms first select candidate regions from the input image and then classify and localize the candidate regions to achieve target detection. These algorithms are represented by R-CNN (region-based convolutional neural network) [13], Fast R-CNN (fast region-based convolutional network) [14], and Faster R-CNN (faster region-based convolutional neural network) [15]. R-CNN combines target region proposal with CNN (convolutional neural network) classification. For the input image, R-CNN first generates candidate regions, uses CNN for feature extraction, and finally uses SVM for classification and box regression. Compared with R-CNN, Fast R-CNN only performs feature extraction for the whole image once, realizing the end-to-end operation of other modules except candidate regions to avoid time wastage. Faster R-CNN adds an RPN network to Fast R-CNN to generate candidate regions, reducing the number of bounding boxes, and changing the whole process into an end-to-end operation. Although Faster R-CNN has good performance and high accuracy, it also has some issues, such as slow processing speed and insensitivity to small targets.

The one-stage target detection algorithm directly obtains the location of the bounding box and the category it belongs to through regression to achieve the detection of targets, and its main algorithms are the SSD [16] and YOLO [17,18,19,20] series. The SSD algorithm was proposed by Liu et al. in 2016. Unlike Faster R-CNN based on candidate region extraction using CNN, SSD uses convolutional networks for direct detection. It detects small targets using feature maps with small receptive fields and large targets using feature maps with large receptive fields and, finally, integrates the detection results into the final prediction. The YOLO family of algorithms eliminates the step of generating candidate regions and implements feature extraction, target classification, and target regression in convolutional neural networks. This simplifies the target detection problem to an end-to-end regression problem, maintaining high accuracy while improving detection speed. Although the detection performance of the YOLO algorithm is good, it still needs to meet the demand for recognition of most object classes in high-speed changes and complex scenes. Therefore, reasonable improvements to the YOLO algorithm in specific areas can improve the detection performance of the target.

## 3. Research Methodology

**Figure 1:**
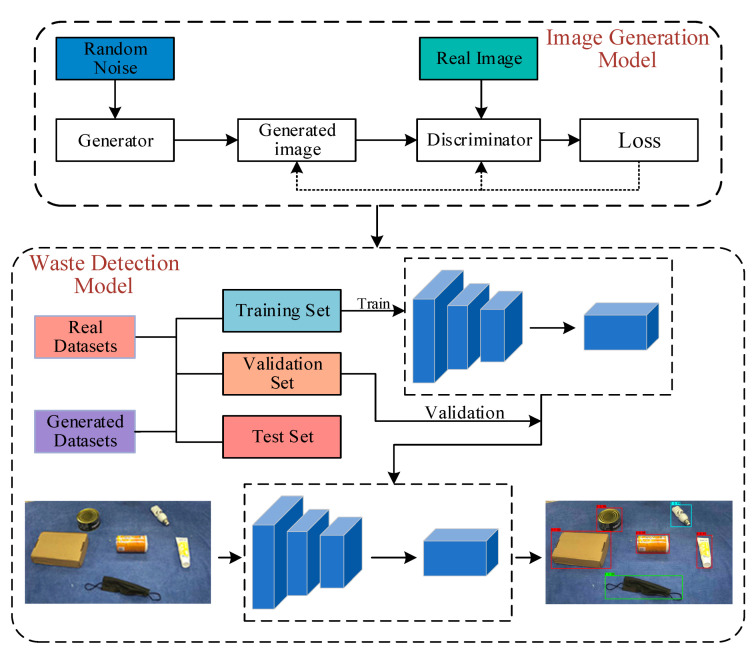
Data Augmentation + YOLO_EC System Flowchart.

The flowchart of the proposed system is presented in Figure 1. Firstly, the sample enhancement strategy based on DCGAN [21] is used to address the issue of insufficient samples from the original data. Next, the generated samples are combined with the real samples to train the target detection model. Finally, the trained model is applied to detect the waste images.

### 3.1. Sample Enhancement Strategy

#### 3.1.1. Framework Structure of DCGAN

**Figure 2:**
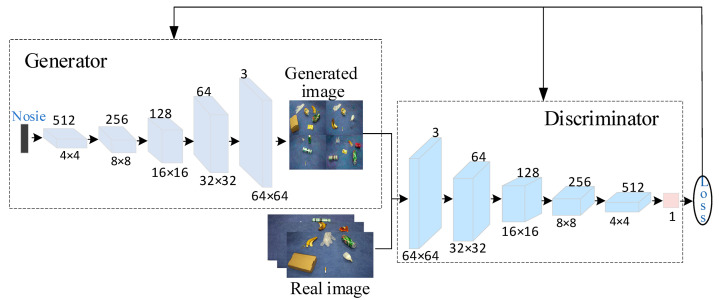
DCGAN network structure.

The deep convolutional generative adversarial network (DCGAN) has a similar structure to GAN. DCGAN employs the convolutional neural network in the GAN model, leveraging the powerful feature extraction capability of CNNs to improve the stability of training and the quality of generated samples. The structure of the DCGAN network is depicted in Figure 2.

First, set a noise *z* that matches the random distribution, and put the noise z into the generator *G* to generate a new image G(z). The objective function of the generator *G* is calculated as shown in Equation (1).

(1) $$L_{G}=E_{z\sim P_{z}(z)}[log(1-D(G(z)))]$$

In Equation (1), $E_{z\sim P_{z}(z)}$ represents the mathematical expectation of the input noise, and D(G(z)) is the discriminator’s probability of discriminating the generated image. The generator’s output image G(z) and real image data $P_{data}(x)$ are fed into the discriminator *D*, which distinguishes the authenticity of the data. The discriminator objective function is calculated as shown in Equation (2).

(2) $$L_{D}=E_{x\sim P_{data}(x)}[log(D(x))]+E_{z\sim P_{z}(z)}[log(1-D(G(z)))]$$

Here, $E_{x\sim P_{data}(x)}$ represents the mathematical expectation of the real image input, and D(x) represents the probability of identifying the real image. The optimization of the objective function during the training process is shown in Equation (3).

(3) $$\begin{array}{ll} F(D,G) & =\underset{G}{\min}\;\underset{D}{\max}V(G,D)\\ & =E_{x\sim P_{data}(x)}[log(D(x{))]+E}_{z\sim P_{z}(z)}[log(1-D(G(z)))] \end{array}$$

The DCGAN network generates images through the generator, aiming to make the discriminator identify errors as the final goal.

#### 3.1.2. Improve DCGAN Network Training Divergence

Although the introduction of convolutional neural networks in DCGAN has improved the network’s performance to some extent, the loss function composition is still based on JS divergence. The problem of gradient disappearance tends to occur during the adversarial training process, resulting in unsatisfactory generated data.

To address this problem, this paper cites Wasserstein distance [22] as a measure of the distance between the generated distribution *P_g_* and the true distribution *P_r_*. The Wasserstein distance is defined as follows:

(4) $$W(P_{r},P_{g})=\underset{\gamma \sim \Pi (P_{r},P_{g})}{\inf}E_{(x,y)\sim \gamma }||x-y||$$

In the equation above, $P_{r}$ represents the true data distribution, $P_{g}$ represents the generated data distribution, $W(P_{r},P_{g})$ denotes the Wasserstein distance between $P_{r}$ and $P_{g}$, “inf” denotes the lower exact bound, $\gamma \sim \Pi (P_{r},P_{g})$ represents the set of joint distributions of $P_{r}$ and $P_{g}$, γ represents one of the joint distributions, x is the true data, and y is the generated data. Additionally, (x,y)~γ indicates that (x,y) is a sample from γ, x−y is the Wasserstein distance between x and y, and $E_{(x,y)\sim \gamma }||x-y||$ is the expectation value of the sample to the distance.

Compared to *JS* divergence, even if there is no intersection between the two distributions of *P_r_* and *P_g_*, the Wasserstein distance still reflects their proximity, as shown in Equations (5) and (6).

(5) $$W(P_{r},P_{g})=|\theta |$$

(6) $$JS(P_{r}||P_{g})=\left\{\begin{array}{cc} \mathrm{Ln}2 & \theta \neq 0\\ 0 & \theta =0 \end{array}\right.$$

From the above formula, it is evident that when two distributions have no intersection, the *JS* divergence value is a constant, and, during gradient descent, the part with a derivative of 0 cannot provide a gradient, leading to gradient disappearance. In contrast, the *W* distance is continuously differentiable, and gradient disappearance does not occur during gradient updates.

Since it is very challenging to compute the *W* distance directly, its dual form is used as follows:

(7) $$W(P_{r},P_{g})=\frac{1}{K}\underset{||f||L\leq K}{\sup}E_{x\sim p_{r}}[f(x)]-E_{x\sim P_{g}}[f(x)]$$

where ||f||L≤K means that the function f(x) satisfies the K-Lipschitz continuity condition. Bringing the *W* distance into the DCGAN network training, a further transformation is achieved as shown in Equation (8).

(8) $$W(P_{r},P_{g})=\underset{D\in 1-\mathrm{Lipschitz}}{\max}\{E_{x\sim P_{data}}[D(x)]-E_{x\sim P_{g}}[D(G(x))]\}$$

where $P_{data}$ denotes the true data distribution; $P_{g}$ denotes the generated data distribution; D∈1-Lipschitz means that the discriminator is to conform to the K-Lipschitz function; D(x) denotes the discriminator output. The objective function of the improved discriminator *D* is shown in Equation (9).

(9) $$obj^{D}=\min\{E_{x\sim P_{g}}[D(G(x))]-E_{x\sim P_{data}}[D(x)]\}$$

As the relationship between generator *G* and discriminator *D* is one of competition (“you gain and I lose”), and $E_{x\sim P_{data}}$ in Equation (8) is independent of the generator, the objective function of the generator can be obtained as follows:

(10) $$obj^{G}=\min\{-E_{x\sim P_{g}}[D(G(x))]\}$$

The introduction of the Wasserstein distance to construct the objective function transforms the original binary classification task of the discriminator network in DCGAN into a regression task. As a result, the sigmoid function needs to be removed from the last layer of the network.

### 3.2. Building YOLO_EC Waste Detection Framework

#### 3.2.1. Network Structure of YOLOv4

**Figure 3:**
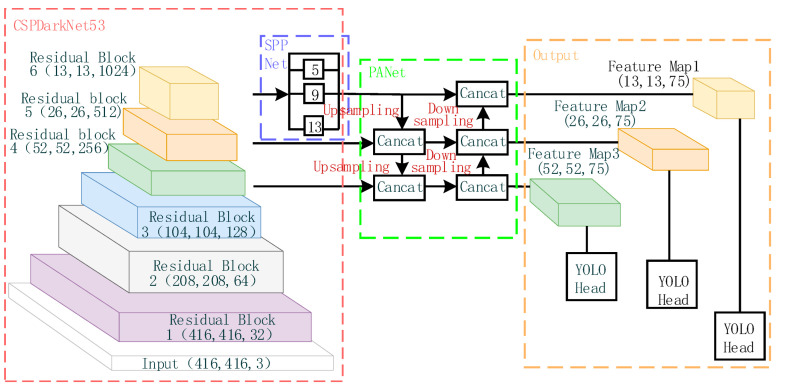
Original YOLOv4 network structure.

YOLOv4 has been widely used by many developers in various engineering fields [23,24,25] and has been proven to have good stability. Additionally, current research has shown that embedding YOLOv4 into different edge devices for detection can achieve good detection results [26,27,28], indicating its high compatibility. In this paper, we use YOLOv4, which is more stable and compatible, as the base algorithm for waste target detection under the premise of satisfying the performance of waste target detection. YOLOv4 primarily comprises three parts: the feature extraction network, the additional module, and the output. The network structure is illustrated in Figure 3. The principle behind the network is to input an image of a fixed size, and, by using regression, determine the location of the bounding box and its corresponding category to achieve target detection.

The backbone feature extraction network of YOLOv4 uses CSPDarknet53, which outputs feature maps with dimensions 52 × 52 × 256, 26 × 26 × 512, and 13 × 13 × 1024 after 8×, 16×, and 32× downsampling. The output feature maps are processed by the additional modules SPP (spatial pyramid pooling) and PANet (pyramid aggregation network). The SPP module operates only on the output feature map of residual block 6, which is pooled by a maximum pooling layer with convolution kernel sizes of 1 × 1, 5 × 5, 9 × 9, and 13 × 13. The results of the pooling are then stacked and outputted. The PAN module upsamples the high-level information to halve the feature map dimension, and stacks it with the upper-level features of the same dimension, while downsampling the low-level information to double the feature map dimension, and stacking it with the lower-level features of the same dimension, achieving feature fusion. Finally, the fused feature maps are input to YOLOhead for decoding and prediction.

#### 3.2.2. EfficientNet Features Extraction Network

The YOLO target detection algorithm predicts objects directly through regression, so selecting an appropriate feature extraction network is critical for detection. In 2019, Google proposed EfficientNet [29], a light weight network that has gained popularity due to its high efficiency and accuracy. In this study, EfficientNet-b2 is chosen as the backbone feature extraction network for YOLOv4, and its network structure is shown in Table 1.

**Figure 4:**
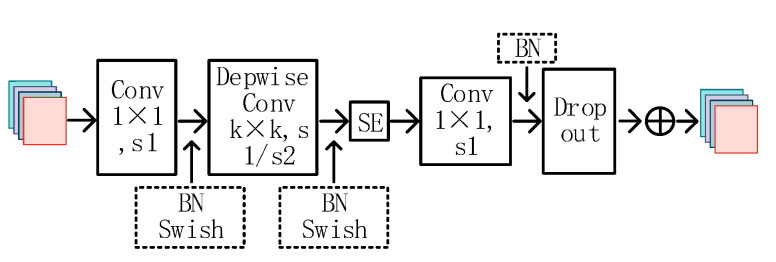
MBConv Module Structure.

According to Table 1, EfficientNet-B2 comprises one stem layer and seven block layers. The stem layer is a convolutional kernel size 3 × 3 convolutional layer. Each block layer is comprised of stacked mobile inverted bottleneck convolutions (MBConv). The structure of the MBConv network is illustrated in Figure 4.

MBConv first uses a 1 × 1 size convolution kernel to change the number of output channels, then performs feature extraction by adding a depth-separable convolution of the SE (squeeze-and-excitation networks) [30] attention module, and, finally, passes through the dropout layer and uses residual connectivity to obtain the final feature map output. The depth-separable convolution reduces the number of model parameters, while the channel attention SE module enhances the representation of salient features. The residual connection is effective in addressing the issue of gradient vanishing caused by excessive network depth.

#### 3.2.3. Refactoring the MBConv Module

**Figure 5:**
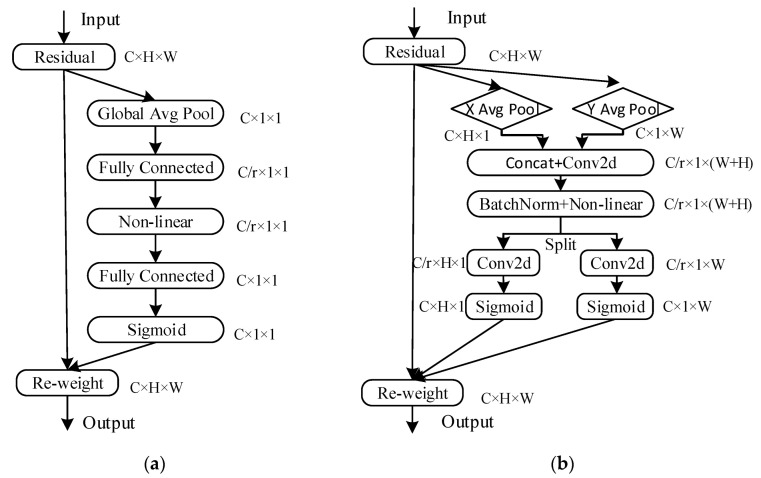
Different attention mechanism module structures: (**a**) SE attention module structure; and (**b**) CA attention module structure.

In recent years, attention mechanisms have been widely utilized in visual detection, resulting in improved performance for convolutional neural networks by enhancing useful feature information and suppressing irrelevant information. To develop more efficient and high-performing networks based on the MBConv module, this study combines the CA (coordinate attention) [31] attention mechanism to replace the SE attention mechanism and reconstructs the MBConv structure for use in the EfficientNet-b2 feature extraction network. The SE and CA attention mechanisms are shown in Figure 5a,b, respectively. The SE attention mechanism only focuses on internal channel information and disregards location information, whereas location information is crucial for acquiring object structure in visual tasks.

To address these shortcomings, the CA attention mechanism incorporates location information into channel attention to avoid the loss of location information caused by two-dimensional global pooling that converts the feature tensor into a single feature vector. For the input feature map X, average pooling kernels with dimensions (H,1) and (1, W) are employed for horizontal and vertical feature extraction, respectively. As a result, two direction-aware feature maps, $Z_{}^{h}$ and $Z_{}^{w}$, are obtained for the horizontal and vertical directions, respectively, with sizes R^C × H × 1^ and R^C × 1 × W^, as demonstrated in Equations (11) and (12).

(11) $$Z_{c}^{h}(h)=\frac{1}{W}\sum_{0\leq i\leq W}X_{c}(h,j)$$

(12) $$Z_{c}^{w}(h)=\frac{1}{H}\sum_{0\leq i\leq H}X_{c}(j,w)$$

The vertical direction-aware feature map $Z_{}^{w}$ is shifted dimensionally to R^C×W×1^. The feature maps with direction-specific information are then concatenated in the third dimension, and the resultant tensor is transformed using the 1 × 1 convolutional transform function $F_{1}$, as depicted in Equation (13).

(13) $$f=\delta (F_{1}([z^{h},z^{w}]))$$

In the equation above, δ represents the nonlinear activation function that generates an intermediate feature map f of size R^C/r × (H+W) × 1^, where r is the proportion of channel downsampling in the convolution. Next, f is split into two feature vectors $f^{h}$ ∈ R^C/r × H × 1^ and $f^{w}$ ∈ R^C/r × 1 × W^ in the channel dimension. These feature vectors are then transformed into the original number of channels using a 1 × 1 convolution. Finally, attention weight maps $g^{h}$ and $g^{w}$ are obtained in two spatial directions using the Sigmoid activation function. These maps exhibit long-range dependence in the feature maps along specific spatial directions, as shown in Equations (14) and (15).

(14) $$g^{h}=\delta (F_{h}(f^{h}))$$

(15) $$g^{w}=\delta (F_{w}(f^{w}))$$

Finally, the input feature map is multiplied by the two weight maps, which enhances the expressiveness of the feature map. The final output of the CA mechanism is shown in Equation (16).

(16) $$y_{c}(i,j)=x_{c}(i,j)\times g_{c}^{h}\left(i\right)\times g_{c}^{w}\left(j\right)$$

#### 3.2.4. YOLO_EC Network Model

**Figure 6:**
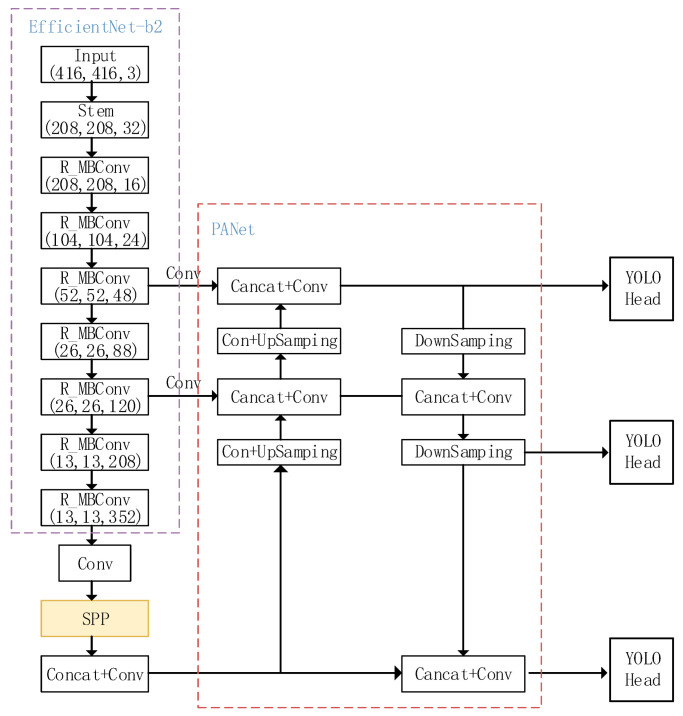
YOLO_EC network structure is proposed in this paper.

For the waste target detection problem, the YOLO_EC target detection model is based on YOLOv4 and incorporates the improvements described in Section 3.2.1, Section 3.2.2 and Section 3.2.3. The resulting model is shown in Figure 6.

We use EfficientNet-b2 as the new feature extraction network, and R_MBConv denotes the MBConv module after refactoring using CA attention in place of the SE attention mechanism. The YOLO_EC network architecture model enables fast detection and classification of waste targets, reducing the complexity of the model while improving its accuracy.

## 4. Experiments and Analysis of Results

### 4.1. Experimental Dataset and Experimental Environment

**Figure 7:**
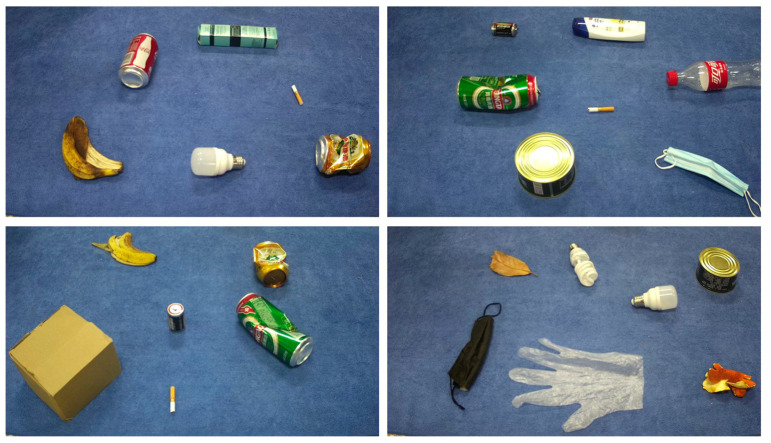
HPU_WASTE dataset image example.

To address the issue of the limited size and single-target type of existing waste datasets, this study introduces a new multi-target household waste dataset named HPU_WASTE. The dataset images were manually captured using the Hikvision DS-2CD3T45D camera, where each image contains multiple waste targets of different types. An example of the HPU_WASTE dataset is illustrated in Figure 7.

The dataset includes four categories of recyclable waste (label 0), other waste (label 1), harmful waste (label 2), and kitchen waste (label 3), covering cigarette butts, batteries (small target sample), and plastic bottles, cans, banana peels (medium target sample) as well as cardboard boxes and disposable gloves (large target sample) of household waste, as shown in Table 2, with a total of 1694 RGB color images.

The HPU_WASTE dataset was used in the experiments. In the data augmentation stage, 1694 acquired photos were used to train the model, resulting in 600 images containing waste targets. In the target detection stage, the HPU_WASTE dataset’s 1694 images were randomly split into a training set (1356 images), a validation set (169 images), and a test set (169 images) using an 8:1:1 ratio. The training set using the data augmentation technique framework consisted of 600 waste target images generated by the modified DCGAN and the training set images of HPU_WASTE; the other comparison methods did not use the data augmentation technique and only used the HPU_WASTE training set. The subsequent comparison experiments were performed on the HPU_WASTE test set.

All experiments were conducted on a graphics workstation equipped with an Intel Xeon E5-2696v4 CPU, an NVIDIA GeForce 3080Ti GPU with 11 GB video memory, and 64 GB of RAM. The PyTorch deep-learning framework and Python programming language were used for implementation. In the data augmentation phase, the parameters were set to a batch size of 32, Adam optimizer, 200 training epochs, and a learning rate of 0.001. In the target detection phase, the input image size was adjusted to 416 × 416, with a batch size of 16, 150 training epochs, and a learning rate of 0.001.

### 4.2. Data Augmentation Experimental Results Analysis

**Figure 8:**
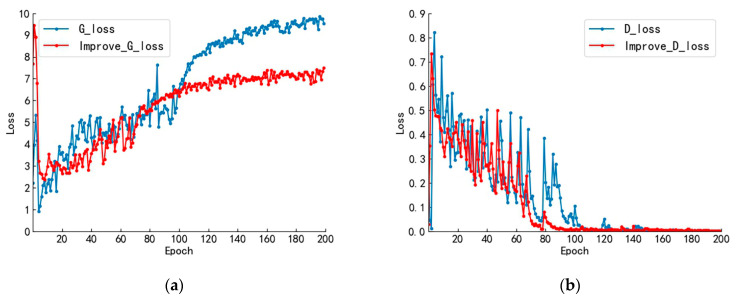
Comparison of DCGAN network loss function change curve before and after improvement: (**a**) comparison of generator loss function variation curves; and (**b**) comparison of discriminant loss function variation curves.

To verify the optimization effect of the improved DCGAN for the training process, we compared the loss function change curves of the original DCGAN network and the W distance improved DCGAN network, as shown in Figure 8. Figure 8a shows the change curve of the generator loss function, while Figure 8b shows the change curve of the discriminator loss function.

In Figure 8a, the generator loss function of the original DCGAN network shows a large fluctuation in the first 100 iterations with an overall increasing trend. After 100 iterations, the model starts to converge with less fluctuation, and the loss function value finally oscillates slightly around 9. Correspondingly, in Figure 8b, the original network discriminator shows an oscillating decay trend in the first 100 iterations, and after 100 iterations, the discriminator stabilizes, and the value of the loss function fluctuates around 0. For the improved network, corresponding to Figure 8a, the generator loss function oscillates upward in the first 80 iterations. After 80 iterations, the model converges, and the oscillation amplitude decreases. The loss function finally oscillates slightly around 7. Corresponding to Figure 8b, the discriminant loss function oscillates decaying in the first 80 iterations. After 80 iterations, the oscillation amplitude decreases, and the value of the loss function fluctuates around 0.

The loss function curves in the training process indicate that both the network structures before and after the improvement have converged and stabilized. This indicates that the generators and discriminators have reached a mutually constrained and balanced state. At the same time, we can also observe the optimization effect of the improved DCGAN on model training, which enhances the stability of the model training and accelerates the convergence speed.

**Figure 9:**
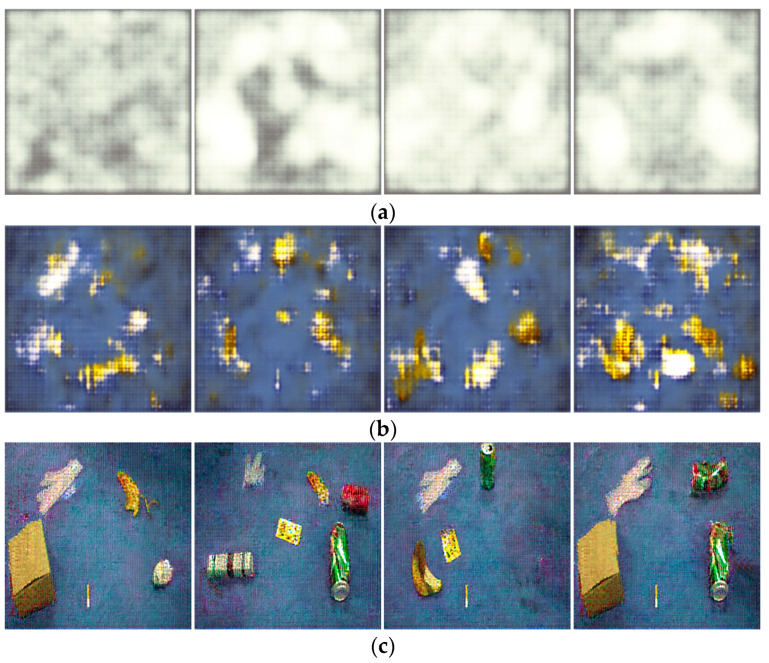
The effect of original DCGAN for generating multi-target waste images under different iterations: (**a**) Epoch10; (**b**) Epoch100; and (**c**) Epoch200.

**Figure 10:**
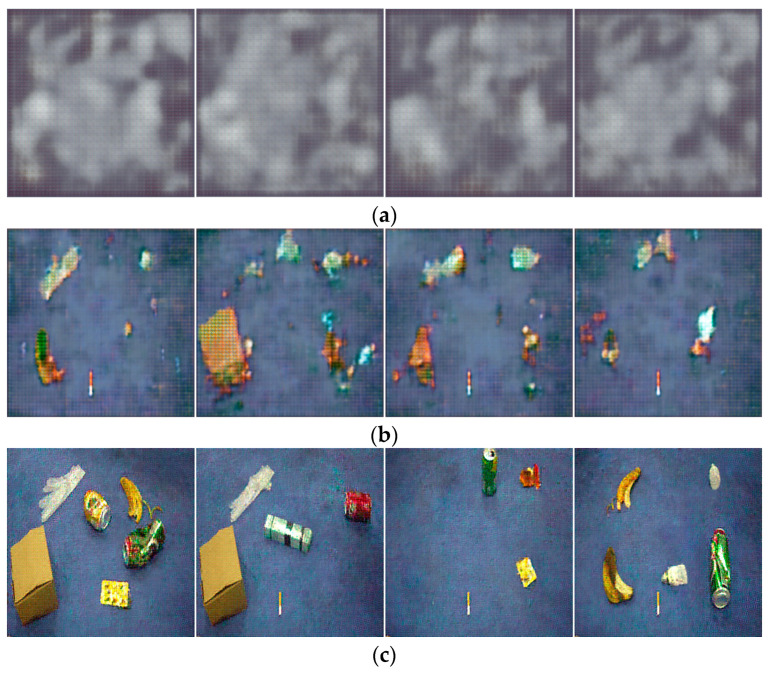
The effect of improved DCGAN for generating multi-target waste images under different iterations: (**a**) Epoch10; (**b**) Epoch100; and (**c**) Epoch200.

To comprehensively study the changes in image generation quality after the improvement, four generated images were randomly selected for display at three stages of model iteration: the early, middle, and late stages, as shown in Figure 9 and Figure 10.

Figure 9 and Figure 10 depict the multi-target waste images generated by the original DCGAN and the improved DCGAN at different numbers of iterations, respectively.

Figure 9 and Figure 10 show that after 10 iterations, the images generated by both models appear distorted and it is almost impossible to recognize any objects. As the number of iterations increases to 100, the generation ability of both networks improves, and the background becomes more similar to real images. However, the original DCGAN still exhibits a grid pattern in its generated images, while the improved model generates images with clearer outlines. When the number of iterations increases to 200, both models generate waste target images, but the improved model generates shapes and backgrounds that are even more similar to real images and with higher image quality. Furthermore, as shown in Figure 9c and Figure 10c, since the noise input to the generator is random, different noise inputs will result in different generated images, making the generative data augmentation method more diverse.

### 4.3. Ablation Experiments

In this paper, a set of ablation experiments is conducted to investigate the impact of different improvements on the YOLOv4 algorithm. The experiments include (1) the original YOLOv4 model, (2) the YOLOv4 model with EfficientNet as the backbone feature extraction network, (3) the YOLOv4 model with EfficientNet+CA as the backbone feature extraction network, (4) the YOLOv4 model using data augmentation, and (5) YOLO_EC (YOLOv4+EfficientNet+CA) with data augmentation. The model evaluation metrics selected are average precision (AP), mean average precision (mAP), model parameters, and frames per second (FPS), and the experimental results are presented in Table 3.

From the experimental results presented in Table 3, it can be observed that using EfficientNet as the backbone feature extraction network reduces the weight of the model to one-fourth of its original size, while greatly improving the detection speed, resulting in an increase in FPS from 14.7 to 24.3. Furthermore, introducing the CA attention mechanism facilitates capturing directional perception and location information while preserving channel information, leading to a significant improvement in the detection of small target objects in other waste and harmful waste. Although the number of model parameters increased by 2.5M, the mean average accuracy (mAP) increased by 3.71% to 94.79%. Additionally, the YOLOv4 model and YOLO_EC model were separately trained using the data augmentation method. Compared with the YOLOv4 and YOLO_EC network frameworks that were not trained with data augmentation, there was a small improvement in AP for different categories, with the mAP increasing by 2.07% and 1.56%, respectively. These results indicate that data augmentation can increase the diversity of features and improve detection accuracy.

Finally, the proposed system in this paper is compared with the original YOLOv4 network, the model size is reduced by 75%, the FPS is improved from 14.7 to 24, and the mean average accuracy mAP is increased by 4.45% to 96.35%, which achieves light weight and improves the detection efficiency of the model at the same time.

**Figure 11:**
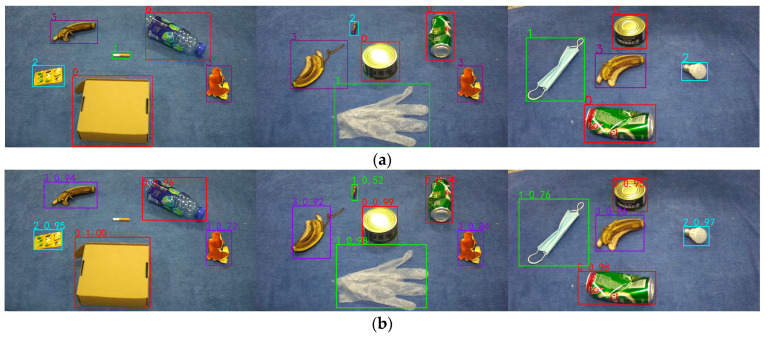

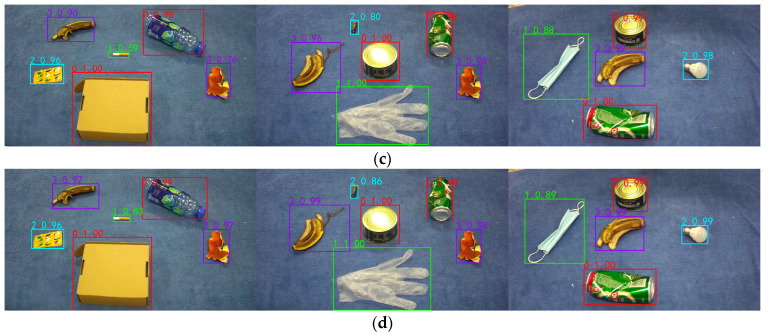
Comparison of model detection effects of different improved methods: (**a**) real label; (**b**) YOLOv4; (**c**) YOLO_EC; and (**d**) data augmentation + YOLO_EC.

Figure 11 shows the comparison plots of the effect of some of the test samples in different detection frameworks. From top to bottom, the labeled graph, the YOLOv4 detection effect graph, the YOLO_EC detection effect graph, and the detection effect graph using data augmentation + YOLO_EC are shown.

As shown in Figure 11, the original YOLOv4 detection framework missed cigarette butts in the first set of experiments and misidentified harmful waste batteries as other waste in the second set of experiments. The improved YOLO_EC detection framework addressed the issue of missed and false detection of small target objects in the first two sets of experiments and also improved the confidence level for detecting medium and large target objects in all three experiments. Using the data augmentation + YOLO_EC detection framework further enhanced the confidence level for target objects and resulted in more accurate localization of the target object.

**Figure 12:**
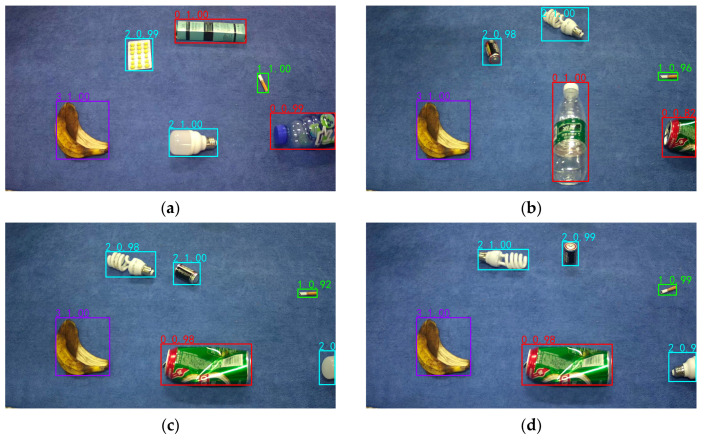

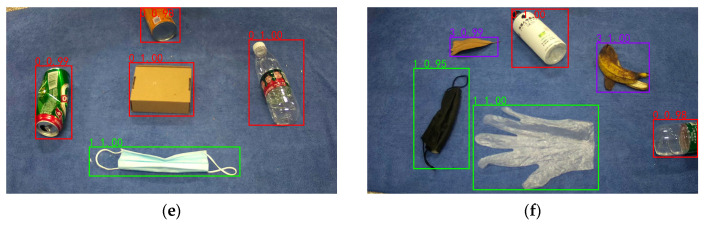
Experimental test results with obscured targets: (**a**) test image 1; (**b**) test image 2; (**c**) test image 3; (**d**) test image 4; (**e**) test image 5; and (**f**) test image 6.

This study conducted experiments on occluded objects to provide a more comprehensive demonstration of the detection performance of data augmentation + YOLO_ES, as shown in Figure 12.

Figure 12 shows that each test image contains occluded objects. In Figure 12a,e, only a small portion of the plastic bottle and metal can are occluded, and the algorithm’s detection precision and object box positioning are very accurate. However, in Figure 12b,f, the occluded area of the recyclable waste metal can and plastic bottle targets exceeds half, and while the occluded plastic bottle detection in Figure 12f still maintains high precision, the accuracy of the metal can detection in Figure 12b is slightly lower than the normal metal can detection. For Figure 12c,d, the occluded area of the harmful waste light bulb target exceeds two-thirds. The occluded object detection accuracy in Figure 12c is only 69%, but the object box positioning is still accurate. The occluded object detection accuracy in Figure 12d reaches 92%, and the object box positioning is very close to the true box. Overall, the YOLO_EC+ data augmentation model proposed in this article has a good detection effect on partially occluded objects, meeting the practical needs of detecting occluded objects.

### 4.4. Comparison with Other Detection Frameworks

To further evaluate the robustness of the proposed framework, we compare the data augmentation + YOLO_EC detection framework with other mainstream detection frameworks, using mAP, model parameters, and FPS as evaluation metrics. The results of the comparison experiments are presented in Table 4.

From Table 4, it can be seen that the data augmentation + YOLO_EC detection framework achieves an increase in mAP of 2.28% compared to the two-stage target detection algorithm Faster R_CNN. However, the Faster R_CNN model is too large in terms of parameters and requires a longer detection time, making it unsuitable for meeting real-time requirements. On the other hand, when compared to the single-stage target detection algorithms YOLOv3, SSD, YOLOv5_L, and YOLOX_L, the proposed algorithm in this paper exhibits significant advantages in all aspects and has a better overall performance.

## 5. Conclusions

In this paper, we use an improved DCGAN to develop a multi-target waste image data augmentation model and use YOLO_EC for light weight and efficient waste object detection. In terms of data augmentation, we introduce the Wasserstein distance as the DCGAN loss function to accelerate model convergence, generate more realistic multi-target waste images, and solve the problem of insufficient data samples and unclear features. In terms of waste object detection, we use EfficientNet-b2 as the YOLOv4 backbone feature extraction network to achieve algorithm light weight and enhance feature extraction by using the CA attention mechanism to reconstruct the MBConv module, thereby improving the model’s detection ability. Experimental results show that the improved DCGAN network has faster training convergence and generates higher-quality images than the original DCGAN with the same number of iterations. The data augmentation + YOLO_EC model compresses the size of the original YOLOv4 network model by 74.05%, improves mAP by 4.54% to 96.35%, and achieves a frame rate of 24 FPS. Compared with other object detection networks, the data augmentation + YOLO_EC model has better robustness and detection performance, with potential practical applications.

The main focus of this research algorithm is the recognition and detection of multi-label waste images on the conveyor belt of a waste processing plant, with shortcomings in target recognition in complex backgrounds and overlapping objects. In future research, the emphasis will be on complex backgrounds and special situations in waste image detection to make it applicable to a wider range of classification scenarios. Additionally, we will combine the target detection framework with the Delta robot [32] to promote the industry’s move towards intelligent development.

## Figures and Tables

**Table 1:** EfficientNet-b2 feature extraction network structure.

Stage	Operator	k × k	Stride	Outputs	Layers
Stem	Conv	3 × 3	2	208 × 208 × 32	1
Black1	MBConv1	3 × 3	1	104 × 104 × 16	2
Black2	MBConv6	3 × 3	2	104 × 104 × 24	3
Black3	MBConv6	5 × 5	2	52 × 52 × 48	3
Black4	MBConv6	3 × 3	2	26 × 26 × 88	3
Black5	MBConv6	5 × 5	1	26 × 26 × 120	4
Black6	MBConv6	5 × 5	2	13 × 13 × 208	5
Black7	MBConv6	3 × 3	1	13 × 13 × 352	2

**Table 2:** HPU_WASTE Dataset Details.

Label	Classes	Category
0	Recyclable waste	Plastic bottles, cans, and cardboard boxes
1	Other waste	Cigarette butts, disposable gloves, and discarded masks
2	Harmful waste	Batteries, light bulbs, and waste drugs
3	Kitchen waste	Banana peel, leaves, and orange peel

**Table 3:** Results of test set ablation experiments.

Model	AP/%				mAP/%	Params/M	FPS
	Recyclable Waste (0)	Other Waste (1)	Harmful Waste (2)	Kitchen Waste (3)			
YOLOv4	95	89	87	96	91.81	256.3	14.7
YOLO+EfficientNet	95	88	87	95	91.08	64	24.3
YOLO+EfficientNet+CA	97	94	91	97	94.79	66.5	24
Data augmentation +YOLOv4	97	92	88	97	93.88	256.3	14.7
Data augmentation +YOLO_EC (YOLO+EfficientNet+CA)	98	96	95	98	96.35	66.5	24

**Table 4:** Comparison of the detection performance of different target detection algorithms.

Model	mAP/%	Params/M	FPS
Faster R_CNN	94.07	547	5.8
YOLOv3	90.03	246.9	13.5
SSD	87.26	105.2	19.6
YOLOv5_L	90.13	187.3	15.6
YOLOX_L	91.27	217.1	15.3
Data augmentation + YOLO_EC	96.35	66.5	24

## Data Availability

The data presented in this study are available on request to the corresponding author with appropriate justification.
